# Editorial Comment to Testicular metastasis from urothelial carcinoma of the bladder

**DOI:** 10.1002/iju5.12407

**Published:** 2021-12-08

**Authors:** Takeshi Yuasa

**Affiliations:** ^1^ Department of Urology Cancer Institute Hospital Japanese Foundation for Cancer Research Ariake Tokyo Japan

Among the clinically organ‐confined genitourinary malignancies, invasive bladder cancer is one of the most lethal diseases, nearly half of which recur even after radical surgery.^1^ For the treatment of recurred metastatic urothelial cancer, therapy with the immune checkpoint inhibitor pembrolizumab is being rapidly introduced in clinical practice.^1^ Enfortumab vedotin, which is a novel antibody drug conjugate anticancer agent, has also very recently been approved as a third‐line standard medical therapy.^2^ The most frequent visceral metastatic site from urothelial cancer is the lung, followed by the liver and bone. Testicular metastasis from bladder cancer is truly rare.

In this issue of *IJU Case Report*, Fukagawa *et al*. reported a case of testicular metastasis from urothelial cancer of the urinary bladder in a patient with a history of holmium laser enucleation of the prostate (HoLEP) for benign prostate hyperplasia and partial penectomy for urethral cancer recurrence after radical cystoprostatectomy.^3^ Three years after the partial penectomy, the patient presented with painless left testicular swelling.^3^ He underwent left high orchiectomy, and histopathological diagnosis disclosed high‐grade urothelial cancer.^3^ In this case, Fukagawa *et al*. supported intraluminal extension via the vas deferens because the patient had a history of HoLEP, which may have opened the ejaculatory duct and allowed malignant cells to penetrate retrogradely and also a history of in situ progression of urothelial cancer to the prostatic duct in the cystectomy specimen.^3^

The majority of testicular tumors are germ cell cancers, which are familiar to urologists in clinical practice. Testicular metastases from other primary sites are rare, accounting for 0.1–2.4% of all testicular tumors.^4^, ^5^ Common primary sites are the prostate (35%), lungs (18%), skin melanoma (11%), colon, and kidneys (9%), which are reported to comprise approximately 80% of primary sites.^4^, ^5^ Although testicular metastasis from bladder cancer is truly rare, in patients with a history of prostatic invasion of bladder cancer and especially in those who also have a history of prostate surgery, clinicians must keep testicular metastasis in mind as a possibility.

## Conflict of interest

The author declares no conflict of interest.
